# Uric Acid Extremes and Lipid Dysregulation: Evidence from a Large Population-Based Study

**DOI:** 10.3390/metabo16070447

**Published:** 2026-06-25

**Authors:** Yazeed Alshuweishi, Ahmed M. Basudan, Zeina S. Alkudmani, Mohammad A. Alfhili

**Affiliations:** Department of Clinical Laboratory Sciences, College of Applied Medical Sciences, King Saud University, Riyadh 12372, Saudi Arabiazalkudmani@ksu.edu.sa (Z.S.A.); malfeehily@ksu.edu.sa (M.A.A.)

**Keywords:** uric acid, hypouricemia, hyperuricemia, lipid profile, dyslipidemia

## Abstract

**Background**: Uric acid (UA) exhibits a dual role, with anti-oxidant or pro-oxidant effects determined by its concentration. However, its association with lipid metabolism across different uric acid states remains unclear. This study explored lipid abnormalities across the full uric acid spectrum in a large adult population. **Methods**: A total of 13,223 adults included in this analysis were classified as hypouricemic, normouricemic, or hyperuricemic based on serum uric acid levels. Lipid profiles, prevalence, associations, risk estimates, and diagnostic accuracy were evaluated using descriptive and multivariate statistical analyses. **Results**: The prevalence of hyperuricemia was 17.26%, while hypouricemia accounted for 2% of the population. Compared with normouricemia, hyperuricemic subjects exhibited substantially greater levels of LDL, TC, and TG and lower HDL concentrations (all *p* < 0.0001). Conversely, hypouricemia exhibited elevated HDL and lower LDL, TC, and TG levels, a pattern consistent across sex and age groups. Lipid abnormalities were most frequent among hyperuricemic participants, notably low HDL (45.9%), high LDL (52.8%), high TC (48.2%), and high TG (36.8%). In contrast, hypouricemia was associated with the lowest prevalence and reduced odds of each abnormality. Serum UA correlated significantly with all lipid measures. In multiple regression analysis, UA remained an independent positive predictor of LDL-C (β = 3.45), TC (β = 3.09), and TG (β = 11.33), and a negative predictor of HDL (β = −2.66) after adjusting for age, sex, glycemia status, and renal function. **Conclusions**: Both UA extremes reflect distinct metabolic states: hyperuricemia showed an adverse lipid profile, whereas hypouricemia was associated with a comparatively more favorable lipid profile, highlighting the association between UA levels and lipid metabolism.

## 1. Introduction

Uric acid, the end product of purine degradation in humans, has complex biological properties and may function as either an anti-oxidant or a pro-oxidant, depending on its concentration and the surrounding cellular environment [1]. Serum uric acid is primarily produced in the liver, intestines and adipose tissue during purine metabolism, with approximately 70% of daily uric acid elimination occurring through renal excretion [1,2]. Serum uric acid levels are regulated by the balance between purine metabolism and renal uric acid excretion [2]. Therefore, hyperuricemia results from either increased uric acid production or impaired renal excretion and may contribute to complications such as gout, tophi formation, nephrolithiasis, and urate nephropathy [1,3,4,5]. Notably, elevated levels of uric acid form a sharp crystal, which deposits in musculoskeletal tissues and leads to gout, the most common inflammatory form of arthritis [3]. Gout has been associated with dyslipidemia, cardiovascular disease, and fatty liver disease [6,7,8], with studies demonstrating a higher prevalence of hypertriglyceridemia among affected patients [9].

Dyslipidemia represents one of the most significant and modifiable contributors to the development of atherosclerosis and cardiovascular diseases [10,11]. The disorder is marked by elevated atherogenic lipids (total cholesterol, LDL, triglycerides) and reduced protective HDL cholesterol [12]. Moreover, it is frequently observed in conjunction with increased adiposity, metabolic dysfunction, and non-alcoholic fatty liver disease (NAFLD), reflecting their shared metabolic underpinnings [13,14,15,16]. It has become more prevalent in most developing countries with the sedentary lifestyle that has come with economic development. During the past two decades, growing attention has been focused on understanding the relationship between hyperuricemia and lipid metabolism. This association extends far beyond the context of gout, emerging as a key metabolic link implicated in a wide range of disorders, including obesity, metabolic syndrome, cardiovascular diseases, and chronic kidney disease. Among these conditions, hyperuricemia has emerged as an important factor associated with increased cardiovascular disease risk, owing to its association with dyslipidemia, hypertension, metabolic syndrome, and atherosclerotic processes [17,18,19].

Several population-based investigations have consistently demonstrated a significant correlation between serum uric acid concentrations and dyslipidemia across diverse adult groups, including those from Bangladesh, Italy, Korea, China, and the United States [20,21,22,23,24]. This relationship appears to transcend ethnic and geographic boundaries, suggesting shared metabolic pathways that link abnormal lipid profiles with elevated serum uric acid levels. While many earlier investigations primarily examined the link between serum uric acid and individual lipid disturbances, especially hypertriglyceridemia, the broader association with the full lipid profile remains less comprehensively explored. Furthermore, a previous study has suggested that hyperuricemia may act as an independent predictor of dyslipidemia in men, whereas this association appears less evident in women [25]. Sex may influence the association between uric acid levels and lipid abnormalities, which merits further consideration. Particularly in Saudi Arabia, there are few studies addressing the link between elevated uric acid and dyslipidemia. Alaql et al. evaluated the association of hyperuricemia with lipid profile parameters among adults, reporting a significant positive correlation between elevated uric acid and TG levels, but no significant link with TC [26]. In contrast, Al-Arfaj et al. found no significant association between uric acid concentration and TG, TC, and HDL levels [27]. Recently, Ghamri et al. demonstrated that higher uric acid concentrations were not associated with lipid profile abnormalities among Saudi adults [28]. Collectively, these findings highlight the complex nature of the relationship between elevated serum uric acid and dyslipidemia, suggesting that it is modulated by demographic, genetic, and metabolic factors. Moreover, various serum uric acid cutoff values have been proposed in the literature, depending on the clinical context and study objectives [29,30]. Nevertheless, most epidemiological and metabolic studies continue to use conventional biochemical and laboratory-based definitions of hyperuricemia [31,32,33]. These classifications are primarily based on laboratory reference intervals and the physiological saturation threshold of monosodium urate in serum. Consequently, biochemical serum uric acid categories remain widely used for metabolic and population-based stratification studies.

Despite these advances, evidence from Middle Eastern populations remains scarce. While hyperuricemia, in particular, has been recently extensively investigated in relation to cardiovascular risk and lipid-derived metabolic indices, including previous studies from our group and others [34,35,36], hypouricemia remains considerably less explored, especially in the context of lipid metabolism and population-based metabolic profiling. Given the high burden of obesity, diabetes, and dyslipidemia in Saudi Arabia, this population provides an important setting for investigating lipid profile alterations across the uric acid spectrum. Accordingly, the primary aim of this study was to characterize the relationship between hypouricemia and lipid abnormalities in a large Saudi population-based cohort while simultaneously comparing these findings with normouricemic and hyperuricemic individuals. This study is, to our knowledge, the first large-scale Saudi investigation to specifically examine lipid profile patterns associated with hypouricemia, thereby providing additional insight into the relationship between lipid alterations and uric acid metabolism.

## 2. Materials and Methods

### 2.1. Study Design and Data Collection

Ethical approval for this retrospective cross-sectional study was obtained from the Biomedical Ethics Unit of Al Borg Diagnostics (Approval No. 07/21; 21 December 2021). Owing to the retrospective design of the study, the requirement for informed consent was waived. Demographic information, including age and sex, together with laboratory measurements such as serum uric acid concentrations and complete lipid profile parameters, were obtained from the Al Borg Diagnostics database. Subjects were categorized according to serum uric acid (SUA) concentrations into hypouricemia (OU; ≤2.6 mg/dL), normouricemia (NU; 2.7–6.8 mg/dL), or hyperuricemia (HU; >6.8 mg/dL). The hyperuricemia threshold (>6.8 mg/dL) was selected because it approximates the physiological saturation point of monosodium urate in serum and is widely used in studies of uric acid metabolism and biochemical hyperuricemia [31,32,33]. As the lower threshold for hypouricemia is not uniformly defined in the literature, the cutoff of ≤2.6 mg/dL was adopted based on the lower limit of commonly reported laboratory reference ranges and epidemiological reports describing serum uric acid distributions [37,38]. Age and sex stratifications followed prior methodologies [39], dividing subjects into young adults (18–39 years), adults (40–64 years), and elderly individuals (≥65 years). Established clinical criteria were used to define dyslipidemia: TC ≥ 200 mg/dL, LDL ≥ 100 mg/dL, HDL < 40 mg/dL, or TG ≥ 150 mg/dL [40].

### 2.2. Statistics

All statistical analyses were performed using GraphPad Prism version 9.2.0, and a two-sided *p* value < 0.05 was considered statistically significant. Continuous variables are presented as medians with interquartile ranges (IQRs). The normality of data distribution was evaluated using the D’Agostino–Pearson and Kolmogorov–Smirnov tests, both of which indicated significant non-normality. Accordingly, non-parametric statistical methods were applied. Comparisons between two independent groups were conducted using the Mann–Whitney U test, whereas differences among three or more groups were assessed using the Kruskal–Wallis test followed by Dunn’s post hoc multiple-comparison analysis. To explore associations between study variables, simple linear regression analyses were initially performed. Thereafter, multiple linear regression models were generated following logarithmic transformation of skewed variables to account for potential confounding effects. Adjustments were made for age, sex, glycemic parameters, and renal function measures. Associations between adjusted study variables were further examined using multivariable logistic regression analysis, from which odds ratios (ORs) and corresponding 95% confidence intervals (CIs) were derived. In addition, receiver operating characteristic (ROC) curve analysis was used to evaluate discriminative performance by calculating the area under the curve (AUC), sensitivity, and specificity.

## 3. Results

### 3.1. Baseline Profile of the Study Population

A total of 13,223 subjects aged ≥18 years were sub-classified according to serum uric acid status into hypouricemia (OU), normouricemia (NU), and hyperuricemia (HU) groups. As summarized in Table 1, several laboratory parameters differed significantly between the uric acid categories. There were higher levels of RBC, WBC, and FBG and lower levels of calcium chloride and potassium in the HU group. Despite the statistical significance of these findings, the absolute differences between groups were relatively modest. Furthermore, the levels of ESR, HbA1c, free T4 and TSH were similar among all groups.

### 3.2. Prevalence of Hyperuricemia in the Studied Population

Among the 13,223 subjects, 265 were hypouricemic (OU), 10,676 normouricemic (NU), and 2282 hyperuricemic (HU). The distribution of serum uric acid categories in the total cohort, as shown in Table 2, revealed prevalences of 2.0% for hypouricemia, 80.7% for normouricemia, and 17.3% for hyperuricemia. The prevalence of hypouricemia decreased slightly across age groups, from 2.0% in young adults to 1.4% in older adults, whereas hyperuricemia was most prevalent among adults (18.0%). Sex-specific analyses showed that hypouricemia was more common among females than males (2.4% vs. 1.4%), while hyperuricemia was more frequent among males than females (20.4% vs. 15.0%).

### 3.3. Sex- and Age-Related Variations in Serum Uric Acid Levels

**Figure 1:**
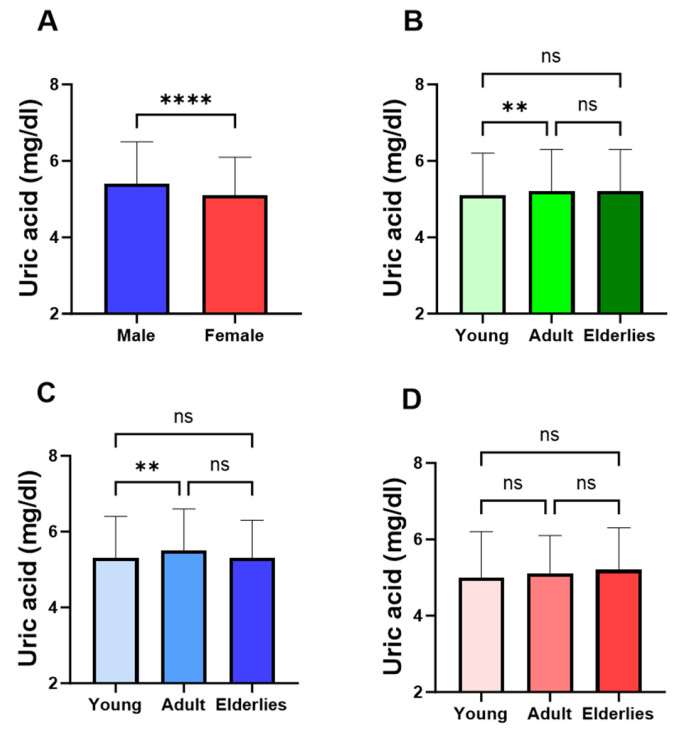
Distribution of serum uric acid levels across demographic subgroups. Panel (**A**) compares median ± interquartile range (IQR) values of serum uric acid between males and females. Panel (**B**) illustrates uric acid levels across age categories for both sexes combined, while Panels (**C**,**D**) display age-related variations within males and females, respectively. Significance levels are denoted by *p* < 0.01 (**) and *p* < 0.0001 (****), and ns is for non-significant differences.

As depicted in Figure 1A, males demonstrated significantly higher serum uric acid concentrations compared with females, although the absolute difference was relatively small (5.40 mg/dL, 5.41–5.50 vs. 5.10 mg/dL, 5.16–5.23). When age groups were analyzed in both genders, the adult group (5.20 mg/dL, 5.30–5.38; Figure 1B) was significantly higher than the young adult group (5.10 mg/dL, 5.23–5.30) but not in elderly individuals (5.10 mg/dL, 5.24–5.41). This pattern was also observed in males (Figure 1C) but not in females (Figure 1D).

### 3.4. Alterations in Lipid Profile Components Across Serum Uric Acid Groups

**Figure 2:**
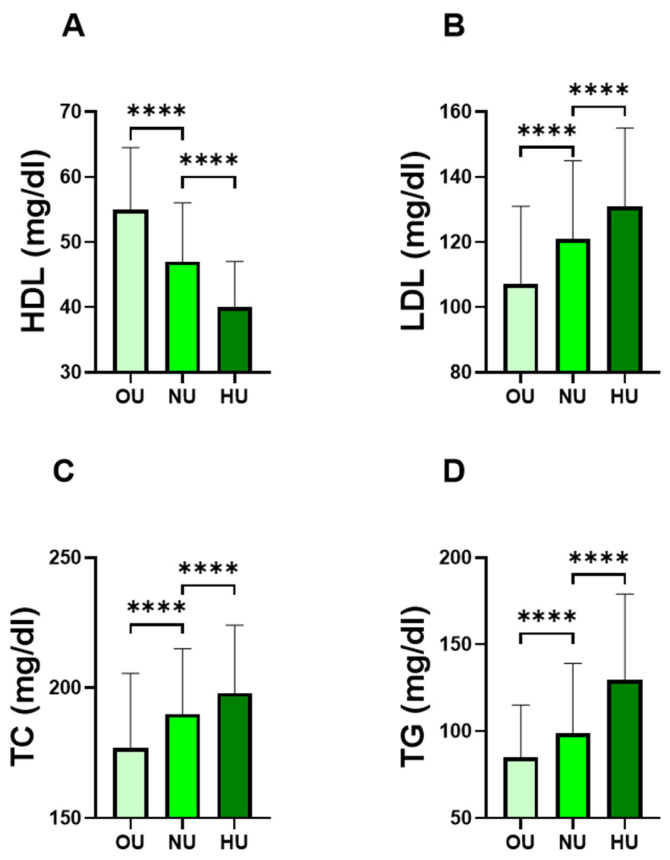
Lipid profile parameters among different uric acid groups in the total study population. Median ± interquartile range (IQR) values are presented for (**A**) HDL, (**B**) LDL, (**C**) total cholesterol (TC), and (**D**) triglycerides (TG) across hypouricemic (OU), normouricemic (NU), and hyperuricemic (HU) groups. Significance levels are represented as *p* < 0.0001 (****), while ns denotes non-significant differences.

Figure 2 displays the distribution of lipid profile parameters among the hypouricemic (OU), normouricemic (NU), and hyperuricemic (HU) groups. Significant differences in individual lipid markers were observed among the uric acid categories. In the OU group, HDL levels were significantly increased (Figure 2A), whereas LDL, TC and TG were substantially decreased (Figure 2B–D) when compared to the NU group. On the other hand, the levels of these lipid components were elevated except for HDL, which was reduced in the HU group (Figure 2A–D).

Similar patterns were observed in sex-stratified analyses, with hyperuricemia generally associated with less favorable lipid profiles in both males and females (Supplementary Figure S1). Moreover, age-stratified analyses demonstrated generally consistent associations between uric acid categories and lipid parameters across different age groups (Supplementary Figure S2). Moreover, an age-wise comparison revealed that HU had elevated LDL, TC, and TG and reduced HDL across all age groups (Supplementary Figure S2), except in elderly individuals, where LDL and TC had a similar level across uric acid groups (Figure 2B,C).

### 3.5. Correlation and ROC Curve Analyses of Serum Uric Acid and Lipid Parameters

**Figure 3:**
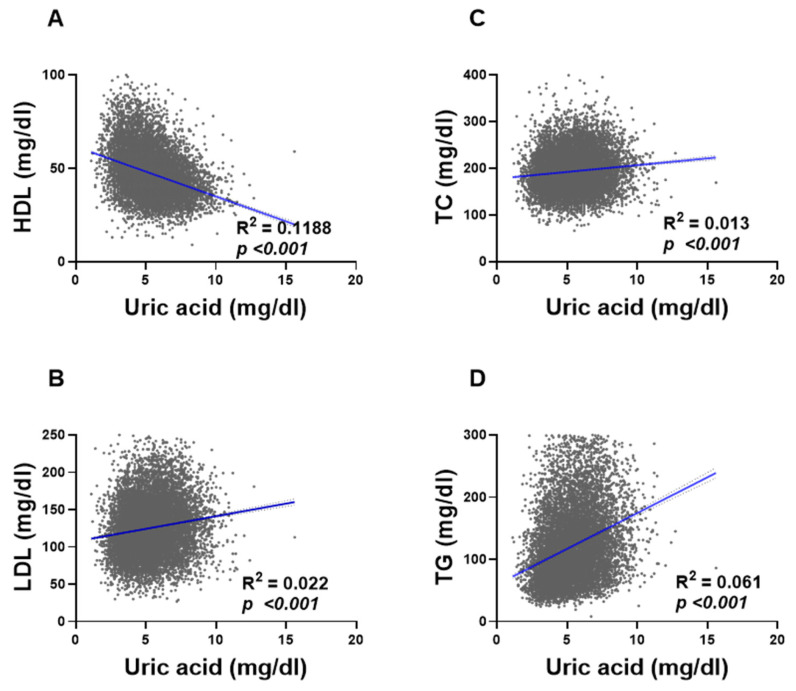
Correlation of lipid markers in predicting hyperuricemia (HU). Panels (**A**–**D**) demonstrate the correlations of serum uric acid with major lipid profile parameters: (**A**) HDL, (**B**) LDL, (**C**) total cholesterol (TC), and (**D**) triglycerides (TG). The blue line illustrates the overall correlation trend between the variables.

Figure 3 depicts the correlations between serum uric acid concentrations and the parameters of the lipid profile in the entire study group. In Figure 3A, HDL demonstrated a significant inverse correlation with serum uric acid concentrations in the overall study population. Although statistically significant, the strength of the association was relatively modest. In ROC curve analysis, HDL showed the highest discriminatory performance among the evaluated lipid parameters for identifying hyperuricemia (Figure S3A; AUC = 0.691), although the predictive ability remained within the modest range. LDL showed a positive correlation with serum uric acid concentrations (Figure 3B); however, the correlation strength was weak. Consistent with this observation, ROC analysis demonstrated limited discriminatory capacity of LDL for identifying hyperuricemia (Figure S3B; AUC = 0.573). Total cholesterol concentrations were also positively associated with serum uric acid levels across the cohort (Figure 3C), yet the observed relationship remained weak. ROC curve analysis further indicated poor-to-modest predictive performance of TC for hyperuricemia detection (Figure S3C; AUC = 0.556). TG levels demonstrated a significant positive correlation with serum uric acid concentrations (Figure 3D). Although stronger than the associations observed for LDL and TC, the relationship remained modest overall. ROC analysis revealed moderate discriminatory performance of TG for identifying hyperuricemia (Figure S3D; AUC = 0.651).

### 3.6. Multivariable-Adjusted Associations of Serum Uric Acid with Lipid Markers

As shown in Table 3, multiple linear regression analyses were conducted to evaluate the association between serum uric acid and lipid profile parameters (HDL, LDL, TC, and TG) across four adjusted models. In the unadjusted model (Model 1), higher uric acid levels were significantly associated with lower HDL (β = −2.65, 95% CI: −2.77 to −2.53) and higher LDL (β = 3.31, 95% CI: 2.94 to 3.69), TC (β = 2.91, 95% CI: 2.49 to 3.33), and TG (β = 11.49, 95% CI: 10.74 to 12.24). After sequential adjustments for demographic factors (Model 2), glycemic parameters (Model 3), and renal function markers (Model 4), the magnitude and direction of these associations remained consistent. In the fully adjusted model, uric acid retained its inverse association with HDL (β = −2.66, 95% CI: −2.82 to −2.50) and positive associations with LDL (β = 3.45, 95% CI: 2.96 to 3.95), TC (β = 3.09, 95% CI: 2.53 to 3.65), and TG (β = 11.33, 95% CI: 10.35 to 12.30).

### 3.7. Occurrence and Risk Evaluation of Lipid Abnormalities Across Serum Uric Acid Status

Table 4 summarizes the prevalence of abnormal lipid markers across the OU, NU, and HU groups. The overall trend demonstrated a progressive increase in lipid abnormalities from OU to HU. Individuals with hypouricemia exhibited the most favorable lipid profile, with low prevalence rates of dyslipidemia—only 13.6% had low HDL, 26.4% had elevated LDL, 29.1% had high total cholesterol, and 8.7% had high triglycerides. In contrast, hyperuricemic subjects showed marked deterioration in lipid parameters: nearly half had low HDL (46.0%), high LDL (52.8%), and high total cholesterol (48.2%), while over one-third (36.8%) had elevated triglycerides. The normouricemic group displayed intermediate values between the two extremes.

Furthermore, adjusted logistic regression models were used to calculate the odds ratios of lipid abnormalities according to serum uric acid categories, with normouricemia as the reference group (Table 5). Hypouricemia was consistently associated with a reduced risk of dyslipidemia, showing significantly lower odds for all lipid abnormalities, including low HDL (adjusted OR = 0.44, 95% CI: 0.27–0.70, *p* < 0.0001), high LDL (adjusted OR = 0.57, 95% CI: 0.41–0.78, *p* < 0.0001), high TC (adjusted OR = 0.60, 95% CI: 0.43–0.84, *p* < 0.0001), and high TG (adjusted OR = 0.27, 95% CI: 0.14–0.48, *p* < 0.0001). Conversely, hyperuricemia demonstrated a strong positive association with adverse lipid profiles. Individuals with elevated uric acid had significantly higher odds of including low HDL (adjusted OR = 2.87, 95% CI: 2.50–3.28, *p* < 0.0001), high LDL (adjusted OR = 1.34, 95% CI: 1.14–1.58, *p* < 0.0001), high TC (adjusted OR = 1.41, 95% CI: 1.24–1.61, *p* < 0.0001), and high TG (adjusted OR = 2.19, 95% CI: 1.90–2.51, *p* < 0.0001).

## 4. Discussion

The present study provides a comprehensive evaluation of lipid abnormalities across different uric acid categories, demonstrating an interconnected relationship between uric acid regulation and lipid metabolic pathways. The findings revealed that hypouricemia was associated with a favorable lipid profile characterized by significantly lower prevalence and reduced odds of lipid abnormalities, including low HDL, high LDL, high total cholesterol, and high triglycerides. In contrast, hyperuricemia was strongly linked to pronounced lipid disturbances, with markedly higher rates and increased odds for all abnormal lipid markers. These findings emphasize that both extremes of uric acid concentration represent distinct metabolic states but with opposing implications: hyperuricemia is accompanied by a higher dyslipidemic burden, while hypouricemia is associated with comparatively more favorable lipid parameters. By studying both hypouricemia and hyperuricemia, it shows contrasting metabolic patterns, more favorable lipid patterns in low uric acid states and adverse lipid patterns in high uric acid states. These findings broaden the understanding of the association between uric acid status and lipid regulation.

In the current study, serum uric acid remained independently associated with triglycerides, total cholesterol, LDL, and HDL after adjustment for demographic, glycemic, and renal parameters. These findings are consistent with several epidemiological studies reporting associations between elevated uric acid and adverse lipid profiles across different populations [41,42]. Moreover, a large cohort study reported that elevated serum uric acid was associated with concurrent dyslipidemia and predicted future development of high triglycerides and low HDL during a 3.5-year follow-up, suggesting a potential temporal relationship [43]. Recent evidence from our group derived from a separate large Saudi cohort study demonstrated that individuals with hyperuricemia exhibited significantly higher levels of several lipid-derived cardiometabolic indices, including triglyceride–glucose (TyG) index, atherogenic index of plasma (AIP), remnant cholesterol (RC), and Castelli risk indices, compared with normouricemic individuals, supporting a close association between elevated uric acid levels and adverse cardiometabolic risk profiles [34]. Collectively, these observations support the association between elevated uric acid and dyslipidemia across diverse populations. Despite the strong statistical associations, the observed AUC values in ROC analyses were generally modest, particularly for LDL-C and total cholesterol, indicating limited discriminative performance and reducing their potential standalone clinical diagnostic utility.

The current study also showed that hypouricemia accounted for 2% of the studied population, representing the first large-scale estimate of hypouricemia prevalence in a Saudi cohort. Previous studies from Korea and Japan reported prevalence rates ranging from approximately 0.2% to 1.4%, depending on population characteristics and genetic background [44,45,46,47]. Notably, none of these studies included a detailed evaluation of lipid profiles in hypouricemic individuals. The present study found that individuals with hypouricemia exhibited comparatively more favorable lipid profiles. Compared with the normouricemic group, hypouricemic individuals had significantly lower prevalence and reduced odds of low HDL, elevated triglycerides, high LDL, and high total cholesterol. This finding suggests that hypouricemia is associated with comparatively favorable lipid parameters in this cohort. Although the association between hypouricemia and lipid markers has been less frequently explored, emerging evidence supports the idea that lower uric acid levels have been associated with more favorable lipid profiles in some studies. A recent network meta-analysis of 13 dietary supplement interventions demonstrated that agents lowering uric acid, such as curcumin and DKB114, also significantly reduced LDL cholesterol levels, supporting a metabolic link between urate regulation and lipid balance [48]. Similarly, a prospective cohort study in pre-dialysis CKD patients showed that allopurinol, a uric acid-lowering drug, independently predicted approximately 13.8% reduction in LDL cholesterol, suggesting a potential association between urate reduction and lipid metabolic changes [49]. Although these reports were conducted in hyperuricemic patients, these findings are consistent with the association observed between lower uric acid levels and comparatively favorable lipid profiles in the present study.

The contrasting lipid profiles observed across uric acid categories suggest a complex relationship between purine and lipid metabolism. Experimental studies have proposed that elevated uric acid may inhibit AMP-activated protein kinase (AMPK), thereby reducing fatty acid oxidation and promoting lipid synthesis [50]. Elevated uric acid has also been associated with activation of SREBP-1c pathways involved in hepatic lipogenesis and lipid accumulation [51]. In adipose tissue, hyperuricemia may contribute to oxidative stress and dysregulated lipolysis, whereas urate-lowering therapy has been reported to improve adipocyte metabolic function [52,53]. In addition, increasing evidence suggests that adiposity and visceral fat accumulation may substantially contribute to the relationship between serum uric acid and dyslipidemia. Epidemiological studies have reported that hyperuricemia is independently associated with visceral adiposity index, central obesity, body roundness index, and weight-adjusted waist index [54,55,56]. Because obesity and visceral adiposity are strongly linked to insulin resistance, altered lipid metabolism, systemic inflammation, and impaired renal urate excretion, residual confounding by adiposity may partly contribute to the associations observed in the current study. Therefore, the absence of BMI, waist circumference, and other anthropometric measures in the present study represents an important limitation, as it limits the ability to determine whether the observed associations were independent of adiposity-related metabolic dysfunction.

These results may contribute to both clinical applications and preventive risk assessment strategies. Since elevated uric acid levels are associated with adverse lipid alterations, monitoring of uric acid levels may aid in the early identification of individuals with elevated metabolic risk. Early lifestyle interventions associated with lower uric acid levels may also contribute to improved lipid parameters. Conversely, although hypouricemia was associated with comparatively favorable lipid profiles in this cohort, these findings should be interpreted cautiously because low uric acid levels may also reflect underlying nutritional, hepatic, renal, or genetic conditions not evaluated in the present study. Moreover, given the large sample size, some statistically significant associations demonstrated relatively modest effect sizes; therefore, their clinical relevance should be interpreted alongside statistical significance. It should also be noted that alternative serum uric acid thresholds associated with cardiovascular morbidity and mortality have been proposed in previous prognostic studies, including the work of Kuwabara et al. and Virdis et al. [29,30]. However, the present study aimed to investigate lipid abnormalities across conventional biochemical uric acid categories rather than to establish prognostic cardiovascular risk thresholds. Future longitudinal studies are warranted to compare conventional biochemical thresholds with prognostic uric acid cutoffs in relation to lipid abnormalities and cardiometabolic outcomes.

The present study has several notable strengths, particularly its large population size, inclusion of participants across the full serum uric acid spectrum, and detailed evaluation of major lipid markers. Nevertheless, certain limitations must be acknowledged. The cross-sectional design of the study limits causal interpretation and prevents determination of the directionality between serum uric acid levels and lipid abnormalities. Furthermore, several potentially important confounding factors were not available in the dataset, including body mass index (BMI)/obesity status, antihyperuricemic therapy, lipid-lowering medications, alcohol intake, smoking status, dietary habits, and physical activity. Given the strong metabolic interrelationship between serum uric acid and lipid metabolism, residual confounding cannot be excluded. Furthermore, the absence of detailed clinical information regarding nutritional status, liver disease, renal tubular disorders, medication use, or genetic causes of hypouricemia limits interpretation of hypouricemia as an inherently favorable metabolic state. Future mechanistic and longitudinal studies should explore the underlying pathways and directionality of this association.

## 5. Conclusions

In summary, this study demonstrated that serum uric acid concentration is closely associated with lipid homeostasis, with distinct metabolic patterns observed across low and high uric acid states. Hyperuricemia was associated with a higher prevalence and greater odds of lipid abnormalities, including elevated LDL, total cholesterol, and triglycerides, along with low HDL, indicating a pronounced dyslipidemic tendency. In contrast, hypouricemia was associated with a lower frequency of lipid abnormalities and reduced odds across lipid markers. These results underscore the relevance of serum uric acid as more than a product of purine catabolism, potentially reflecting alterations in lipid metabolism and overall metabolic condition. Integrating uric acid evaluation into routine biochemical assessments may help identify individuals at risk for lipid disturbances and support ongoing investigations into the metabolic implications of serum uric acid.

## Figures and Tables

**Table 1:** Summary of subject variables and laboratory parameters.

Characteristics	OU (*n* = 265)	NU (*n* = 10,676)	HU (*n* = 2282)	*p*-Value
Age (years)	39 (40.22–43.36)	39 (42.46–42.99)	40 (42.48–43.65)	0.32
Male (%)	0.58	32.39	8.45	
Female (%)	1.42	48.35	8.80	
RBCs (×10^6^/μL)	4.95 (4.97–5.10)	5.18 (5.21–5.23)	5.65 (5.59–5.64)	<0.0001
WBCs (×10^6^/μL)	5.49 (5.61–6.10)	5.72 (5.92–6.00)	6.09 (6.25–6.41)	<0.0001
ESR (mm/h)	10.00 (13.13–17.59)	8.00 (12.03–12.62)	8.00 (11.58–13.39)	0.08
FBG (mg/dL)	93 (110.9–127.7)	94 (103.9–105.4)	98 (104.6–107.5)	<0.0001
HbA1c (%)	5.5 (5.79–7.20)	5.5 (5.99–6.08)	5.6 (5.86–6.01)	0.15
Free T4 (ng/dL)	1 (1.00–1.06)	1 (1.00–1.02)	0.99 (1.00–1.06)	0.10
TSH (mIU/L)	1.87 (2.02–3.00)	1.79 (2.42–2.57)	1.725 (2.24–2.53)	0.07
Ca (mg/dL)	9.6 (9.56–9.67)	9.6 (9.63–9.65)	9.6 (9.58–9.61)	<0.0001
Cl (mEq/L)	106 (104.5–105.8)	104 (104.1–104.3)	105 (104.6–105.0)	<0.0001
K (mEq/L)	4.3 (4.27–4.40)	4.4 (4.42–4.45)	4.3 (4.33–4.38)	<0.0001

Results are shown as median ± IQR. RBC, red blood cell; WBC, white blood cell; ESR, erythrocyte sedimentation rate; FBG, fasting blood glucose; TSH, thyroid-stimulating hormone; Ca, calcium; Cl, chloride; K, potassium.

**Table 2:** Prevalence of uric acid categories according to age and sex.

	OU (%)	NU (%)	HU (%)
Overall	2.0	80.7	17.3
Age			
young adult (*n* = 6630)	2.0	81.3	16.6
Adult (*n* = 5399)	1.8	80.1	18.0
elderly individuals (*n* = 1179)	1.4	81.1	17.5
Gender			
Male (*n* = 5478)	1.4	78.2	20.4
Female (*n* = 7745)	2.4	82.5	15.0

Abbreviations: OU = hypouricemia; NU = normouricemia; HU = hyperuricemia.

**Table 3:** Association between serum uric acid and lipid profile components: multivariable linear regression analysis.

Model	HDL (β, 95% CI)	LDL (β, 95% CI)	TC (β, 95% CI)	TG (β, 95% CI)
Model 1	−2.65 [−2.77–−2.53]	3.31 [2.94–3.69]	2.91 [2.49–3.33]	11.49 [10.74–12.24]
Model 2	−2.63 [−2.76–−2.51]	3.30 [2.92–3.67]	2.92 [2.50–3.34]	11.44 [10.69–12.19]
Model 3	−2.55 [−2.74–−2.36]	3.41 [2.81–4.01]	3.09 [2.43–3.75]	11.89 [10.66–13.12]
Model 4	−2.66 [−2.82–−2.50]	3.45 [2.96–3.95]	3.09 [2.53–3.65]	11.33 [10.35–12.30]

β represents regression coefficients. Model 1: unadjusted; Model 2: adjusted for age and sex; Model 3: adjusted for age, sex, FBG, and HbA1c; Model 4: adjusted for age, sex, creatinine, and urea. All associations were statistically significant (*p* < 0.0001).

**Table 4:** Distribution of dyslipidemia components by uric acid status.

Parameter	OU	NU	HU
Normal HDL	86.42	77.80	54.03
Low HDL	13.58	22.20	45.97
Normal LDL	73.58	59.54	47.20
High LDL	26.42	40.46	52.80
Normal TC	70.94	60.31	51.80
High TC	29.06	39.69	48.20
Normal TG	91.32	79.69	63.19
High TG	8.68	20.31	36.81

Values are expressed as percentages. OU = hypouricemia; NU = normouricemia; HU = hyperuricemia; HDL = high-density lipoprotein cholesterol; LDL = low-density lipoprotein cholesterol; TC = total cholesterol; TG = triglycerides.

**Table 5:** Adjusted odds ratios for lipid abnormalities according to serum uric acid category.

	Hypouricemia			Hyperuricemia		
Lipid Abnormality	Odds Ratios	95% CI	*p* Value	Odds Ratios	95% CI	*p* Value
Low HDL	0.44	0.27–0.70	<0.0001	2.87	2.50–3.28	<0.0001
High LDL	0.57	0.41–0.78	<0.0001	1.34	1.14–1.58	<0.0001
High TC	0.60	0.43–0.84	<0.0001	1.41	1.24–1.61	<0.0001
High TG	0.27	0.14–0.48	<0.0001	2.19	1.90–2.51	<0.0001

Abbreviations: HDL, high-density lipoprotein cholesterol; LDL, low-density lipoprotein cholesterol; TC, total cholesterol; TG, triglycerides. Adjusted logistic regression analyses were constructed to estimate the odds ratios for lipid abnormalities across serum uric acid categories after adjustment for potential confounding factors, including age, sex, glucose metabolic status, and renal function. Normouricemia served as the reference category.

## Data Availability

Access to the data presented in this study is available upon reasonable request to the corresponding author, subject to institutional approval.

## Supplementary Materials

The following supporting information can be downloaded at: https://www.mdpi.com/article/10.3390/metabo16070447/s1, Figure S1. Lipid profile parameters across different uric acid groups in males and females. Median ± interquartile range (IQR) values of (A) HDL, (B) LDL, (C) total cholesterol (TC), and (D) triglycerides (TG) are shown separately for males and females classified as hypouricemic (OU), normouricemic (NU), or hyperuricemic (HU). Significance levels are represented as *p* < 0.001 (***) and *p* < 0.0001 (****), whereas ns denotes non-significant differences. Figure S2. Lipid profile parameters across different uric acid groups stratified by age category. Median ± interquartile range (IQR) values of (A) HDL, (B) LDL, (C) total cholesterol (TC), and (D) triglycerides (TG) are shown for hypouricemic (OU), normouricemic (NU), and hyperuricemic (HU) groups within three age categories: young adults (18–39 years), adults (40–64 years), and elderlies (≥65 years). Significance levels are represented as *p* < 0.05 (*), *p* < 0.01 (**), *p* < 0.001 (***) and *p* < 0.0001 (****), whereas ns denotes non-significant differences. Figure S3. Diagnostic performance of lipid markers in predicting hyperuricemia (HU). Panels (A–D) present receiver operating characteristic (ROC) curve analyses evaluating the ability of these lipid markers to discriminate hyperuricemic (HU) from normouricemic (NU) individuals.
