# Supplementary material for: Uric Acid Extremes and Lipid Dysregulation: Evidence from a Large Population-Based Study
Source: Metabolites. 2026 Jun 25;16(7):447. doi: 10.3390/metabo16070447 (PMC13413473; doi:10.3390/metabo16070447)
Supplement: Supplementary file 1 [file metabolites-16-00447-s001.zip › metabolites-4303798-supplementary.pdf]

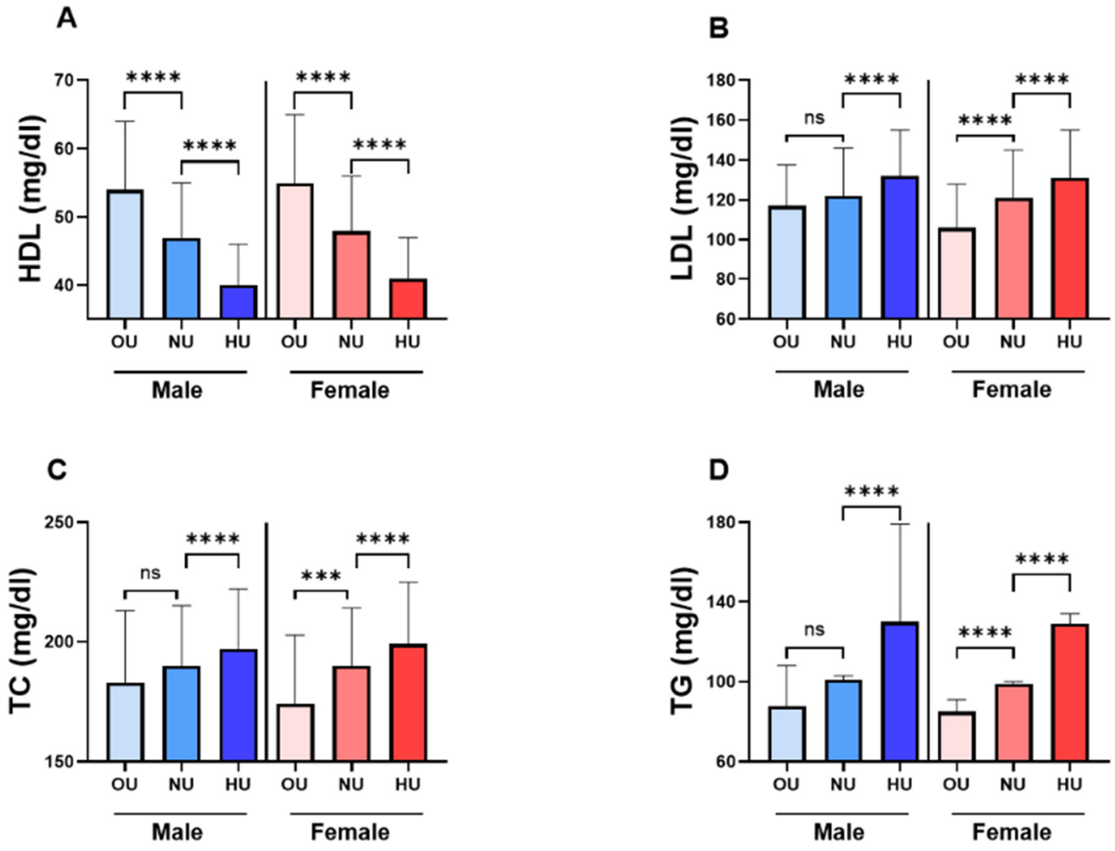

Figure S1. Lipid profile parameters across different uric acid groups in males and females. Median ± interquartile range (IQR) values of (**A**) HDL, (**B**) LDL, (**C**) total cholesterol (TC), and (**D**) triglycerides (TG) are shown separately for males and females classified as hypouricemic (OU), normouricemic (NU), or hyperuricemic (HU). Significance levels are represented as $p < 0.001$ (***) and $p < 0.0001$ (****), whereas ns denotes non-significant differences.

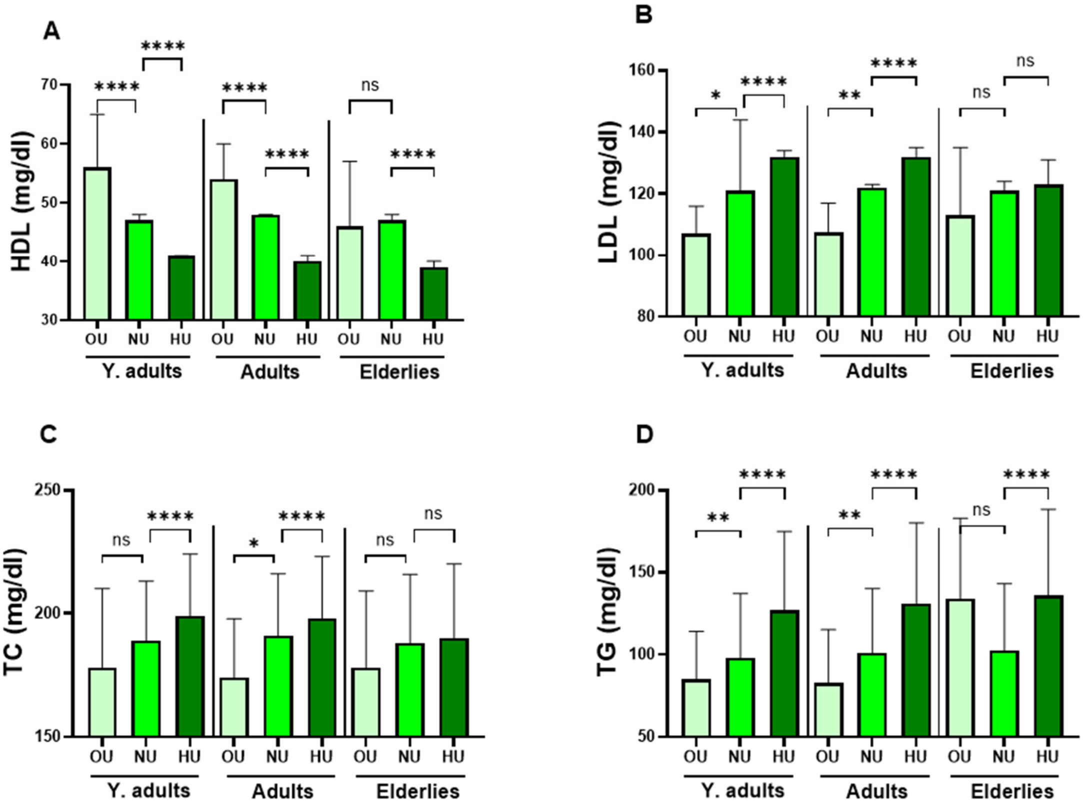

Figure S2. Lipid profile parameters across different uric acid groups stratified by age category. Median ± interquartile range (IQR) values of (**A**) HDL, (**B**) LDL, (**C**) total cholesterol (TC), and (**D**) triglycerides (TG) are shown for hypouricemic (OU), normouricemic (NU), and hyperuricemic (HU) groups within three age categories: young adults (18–39 years), adults (40–64 years), and elderlies (≥65 years). Significance levels are represented as $p < 0.05$ (*), $p < 0.01$ (**), $p < 0.001$ (***) and $p < 0.0001$ (****), whereas ns denotes non-significant differences.

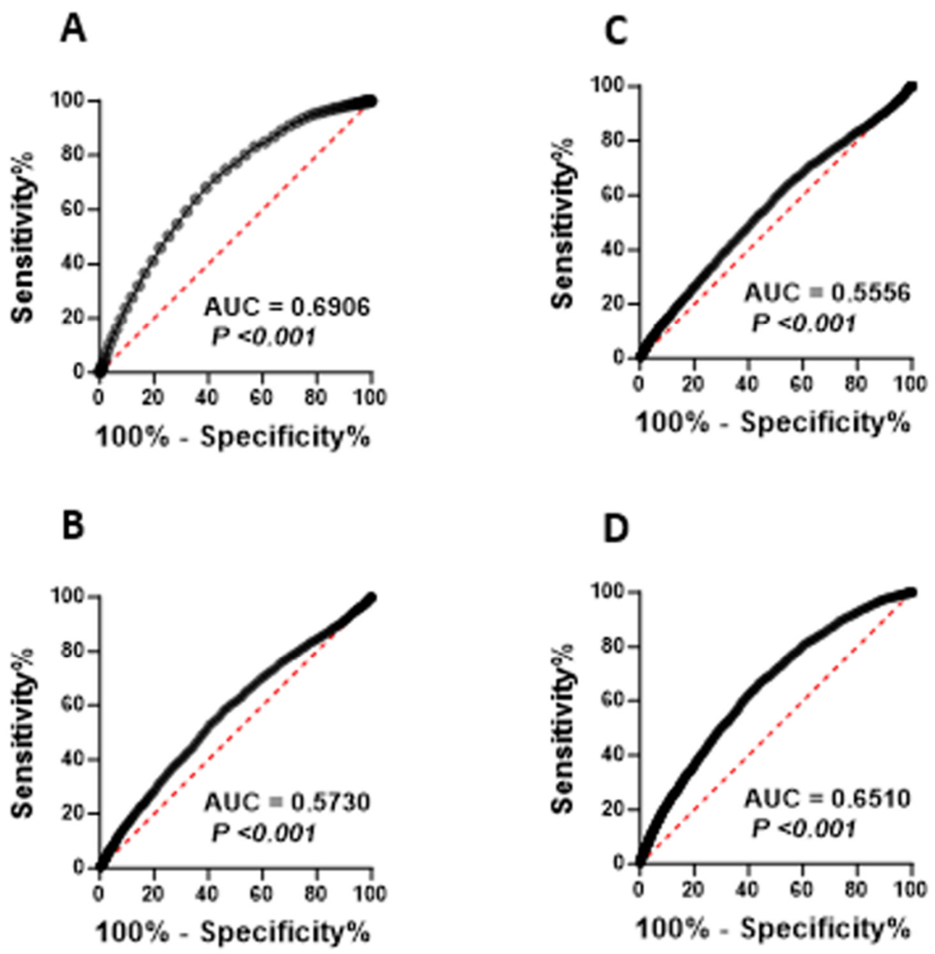

Figure S3. Diagnostic performance of lipid markers in predicting hyperuricemia (HU). Panels (**A**–**D**) present receiver operating characteristic (ROC) curve analyses evaluating the ability of these lipid markers to discriminate hyperuricemic (HU) from normouricemic (NU) individuals.
